# The arabinose transporter MtLat-1 is involved in hemicellulase repression as a pentose transceptor in *Myceliophthora thermophila*

**DOI:** 10.1186/s13068-023-02305-3

**Published:** 2023-03-25

**Authors:** Shuying Gu, Zhen Zhao, Fanglei Xue, Defei Liu, Qian Liu, Jingen Li, Chaoguang Tian

**Affiliations:** 1 Key Laboratory of Engineering Biology for Low-carbon Manufacturing, Tianjin Institute of Industrial Biotechnology, Chinese Academy of Sciences, Tianjin, 300308 China; 2 National Technology Innovation Center of Synthetic Biology, Tianjin, 300308 China; 3 University of Chinese Academy of Sciences, Beijing, 100049 China; 4 College of Biotechnology, Tianjin University of Science and Technology, Tianjin, 300457 China

**Keywords:** *Myceliophthora*, Hemicellulase, l-Arabinose transport, Signal transduction, Transceptor, MtLat-1, MtAra-1

## Abstract

**Background:**

Filamentous fungi possess an array of secreted enzymes to depolymerize the structural polysaccharide components of plant biomass. Sugar transporters play an essential role in nutrient uptake and sensing of extracellular signal molecules to inhibit or trigger the induction of lignocellulolytic enzymes. However, the identities and functions of transceptors associated with the induction of hemicellulase genes remain elusive.

**Results:**

In this study, we reveal that the l-arabinose transporter MtLat-1 is associated with repression of hemicellulase gene expression in the filamentous fungus *Myceliophthora thermophila*. The absence of *Mtlat-1* caused a decrease in l-arabinose uptake and consumption rates. However, mycelium growth, protein production, and hemicellulolytic activities were markedly increased in a Δ*Mtlat-1* mutant compared with the wild-type (WT) when grown on arabinan. Comparative transcriptomic analysis showed a different expression profile in the Δ*Mtlat-1* strain from that in the WT in response to arabinan, and demonstrated that MtLat-1 was involved in the repression of the main hemicellulase-encoding genes. A point mutation that abolished the l-arabinose transport activity of MtLat-1 did not impact the repression of hemicellulase gene expression when the mutant protein was expressed in the Δ*Mtlat-1* strain. Thus, the involvement of MtLat-1 in the expression of hemicellulase genes is independent of its transport activity. The data suggested that MtLat-1 is a transceptor that senses and transduces the molecular signal, resulting in downstream repression of hemicellulolytic gene expression. MtAra-1 protein directly regulated the expression of *Mtlat-1* by binding to its promoter region. Transcriptomic profiling indicated that the transcription factor MtAra-1 also plays an important role in expression of arabinanolytic enzyme genes and l-arabinose catabolism.

**Conclusions:**

*M. thermophila* MtLat-1 functions as a transceptor that is involved in l-arabinose transport and signal transduction associated with suppression of the expression of hemicellulolytic enzyme-encoding genes. The data presented in this study add to the models of the regulation of hemicellulases in filamentous fungi.

**Supplementary Information:**

The online version contains supplementary material available at 10.1186/s13068-023-02305-3.

## Background

Non-edible plant biomass is recognized as a potential sustainable source for production of biofuels and commodity chemicals in biorefinery processes [1]. However, the costs involved in conversion of insoluble plant lignocellulose into fermentable sugar remain a significant barrier to commercialization [2]. Filamentous fungi are able to secrete a series of hydrolytic enzymes to synergistically deconstruct plant polysaccharides. Several fungi, such as *Trichoderma reesei*, *Myceliophthora thermophila*, and *Penicillium oxalicum*, have been developed as the platforms to produce cellulolytic enzymes for industrial applications [3–5].

In fungi, lignocellulolytic enzyme-encoding genes are regulated by the complex signaling networks. Sugar transporters play an essential role in substrate uptake and sensing extracellular signal molecules to inhibit or trigger enzyme induction [6–8]. The transporter with signaling activity is called as transceptor. In *Neurospora crassa*, cellodextrin transporters CDT-1 and CDT-2, and cellobionic acid transporter CLP1 are involved in the induction of expression and secretion of cellulases. The function of CDT-1 and CDT-2 on signal transduction for the activation of cellulolytic gene expression is not dependent on the transporting activities [8–10]. In *T. reesei*, a dual cellobiose/glucose transporter Stp1 is involved in the carbon catabolite repression response (CCR) and repressed induction of cellulase and hemicellulase genes [7]. Similarly, glucose high-affinity transporters HGT-1/2 inhibit cellulase gene expression by both CCR and the cyclic adenosine monophosphate-protein kinase A pathway in *N. crassa* [6]. Some proteins that possess multiple transmembrane domains but have lost the capacity for sugar transport, also serve as sugar sensors, such as *Saccharomyces cerevisiae Snf3* and *Rgt2* [11], and *N. crassa* RCO3 [12, 13]. Snf3 and Rgt2 sense extracellular low and high glucose levels, respectively, and transduced molecular signal with cytoplasmic C-terminal tail to mediate a glucose signaling pathway [14]. However, the transceptor involved in induction of hemicellulase genes remains to be identified and characterized.

Fungal cellulase and hemicellulase gene expression is tightly regulated at the transcriptional level upon appropriate nutrient sensing [15]. Several essential transcription factors have been studied, mostly in saprobic fungi, including CreA/CRE-1, XYR1 (XLR-1/XInR), CLR-1/A, and CLR-2/B (ManR) [15–19]. CLR-2 and its orthologs are the major regulators of genes encoding cellulolytic enzymes in *N. crassa*, *Aspergillus nidulans*, and *P. oxalicum* [18–20]. Transcription factors associated with hemicellulose deconstruction include Xyr1/XlnR, CreA/CRE-1, and Ara-1. The transcriptional activator XlnR/XYR1 is a major positive activator of the expression of hemicellulase genes in a number of filamentous fungi, although the enzymes that are actually regulated by it differ in different species. In *T. reesei* and *Aspergillus niger* [21–23], Xyr1/XlnR regulates both cellulase and hemicellulase genes, whereas in species such as *N. crassa*, *M. thermophila*, and *Fusarium graminearum*, XlnR homologs regulate xylanase expression and d-xylose utilization [16, 24, 25]. Similarly, in *Fusarium oxysporum*, a strain carrying a deletion of *xlnR* lacked transcriptional activation of structural xylanase genes and exhibited dramatically reduced extracellular xylanase activity [26]. CreA/CRE-1 not only inhibits the expression of the major hemicellulase/cellulase genes, but also suppresses key sugar catabolic genes and sugar transporters in *N. crassa* and *Aspergillus *spp. [27, 28]. In addition, another transcription factor, Ara1, modulates the use of arabinose and arabinan in *N. crassa* [27], *Magnaporthe oryzae* [29] and *T. reesei* [14], while a different transcription factor (AraR) functions in an analogous manner in *A. niger* [30].

*Myceliophthora thermophila* is a thermophilic fungus known for its capability to efficiently secrete a complete set of thermostable carbohydrate-active enzymes [31, 32]. Recently, this fungus has been developed as an important platform for the production of industrial enzymes [4] and as a cell factory to produce biochemicals and biofuels by directly conversing plant biomass [5, 33–35]. A few lignocellulose degradation-related transcription regulators have been identified and characterized in this fungus, including XYR1, CRE-1, CLR-1/2, and CLR-4 [25, 36, 37]. We previously characterized the l-arabinose transporter MtLat-1 and demonstrated that MtLat-1 exhibits specific transport activity for l-arabinose [38]. In the present study, we investigated the role of MtLat-1 in the repression of hemicellulase genes of *M. thermophila*. On the basis of comparative transcriptomic analysis and the assays of a non-transporting MtLat-1 mutant, we found that MtLat-1 functions as a transceptor, which is involved in l-arabinose transportation, sensing, and downstream signaling cascades in *M. thermophila*. In addition, we also demonstrated that *Mtlat-1* expression is directly regulated by transcription factor MtAra-1, which was involved in l-arabinose release and metabolism.

## Results

### The role of sugar transporter MtLat-1 in the growth of ***M. thermophila*** on l-arabinose

**Fig. 1:**
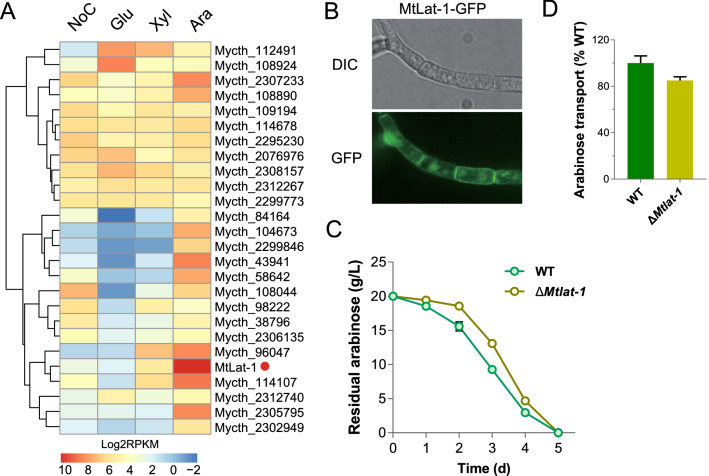
Physiological characterizations of *M. thermophila* with deletion of *Mtlat-1* in response to l-arabinose. **A** Heatmap analysis of the expression of 26 sugar transporters with robust expression levels (RPKM > 20) in at least one tested condition (NoC, no carbon; Glu, d-glucose; Xyl, d-xylose; Ara, l-arabinose). Log-transformed expression values are color-coded. **B** Subcellular localization of MtLat-1 in *M. thermophila*. **C** Sugar consumption of strain Δ*Mtlat-1* when grown in 1 × Vogel’s minimal medium (VMM) with 2% l-arabinose. **D** l-Arabinose transport rates of mycelia from the Δ*Mtlat-1* mutant. Error bars indicate the standard deviation (SD) from at least three biological replicates

Our previous study revealed that sugar transporter MtLat-1 (Mycth_95427) showed a high uptake activity and specificity for l-arabinose [38]. To determine the induction of *Mtlat-1* on various carbon sources, we conducted comparative transcriptomic analysis of *M. thermophila* grown on plant biomass-derived monosaccharides, including d-glucose, d-xylose, and l-arabinose. When responding to l-arabinose, 26 putative sugar transporter genes (54% of the total sugar transporter genes in *M. thermophila*) showed high expression levels (RPKM > 20), including five putative glucose transporter genes (Mycth_112491, Mycth_2308157, Mycth_2295230, Mycth_109194, Mycth_108044), four putative glucose and pentose transporter genes (Mycth_2305795, Mycth_96047, Mycth_108924, Mycth_2302949), and two putative cellodextrin transporter genes (Mycth_114107 and Mycth_43941) (Fig. 1a and Additional file 1: Table S3), similar to observations in *N. crassa,* where the major sugar transporter genes were highly induced by l-arabinose [39]. Consistent with its substrate specificity, dramatic induction of MtLat-1 (RPKM > 1000) was only observed on l-arabinose. In addition, previous kinetic property assay revealed that MtLat-1 has a great maximum velocity (*V*_max_) of l-arabinose uptake. Therefore, we were interested in the role of the MtLat-1 in the growth of *M. thermophila* on plant biomass-derived monosaccharides.

The *Mtlat-1*-null strain was constructed via homologous replacement with a neomycin resistance gene (*neo*)-inclusive cassette using CRISPR/Cas9 system [40]. The correct deletion and recombination events in the resulting mutant were confirmed by PCR. When grown on d-glucose or the three most relevant hemicellulose side-chain sugars (d-xylose, l-arabinose, and d-galactose), the consumption rates of d-glucose, d-xylose, and d-galactose in the Δ*Mtlat-1* mutant were similar to those of the wild-type (WT) strain (Additional file 2: Fig. S1), but a clear retardation of l-arabinose consumption was observed in culture of strain Δ*Mtlat-1* (Fig. 1). To support these data, we conducted sugar uptake assays using the mycelia pre-induced by l-arabinose. The l-arabinose concentration in the culture supernatants was measured over time. Analysis of the initial uptake rate (20 min) indicated that l-arabinose uptake was reduced by approximately 15% in the Δ*Mtlat-1* mutant compared with that in the WT (15.4 nmol/min/mg_DCW) (Fig. 1d). In the *lat-1* deletion strain of *N. crassa*, l-arabinose uptake was almost completely abolished [41]. However, the deletion of MtLat-1 ortholog (Trire2_104072) in *T. reesei* did not cause an altered growth phenotype at high (1%) l-arabinose level, but led to a growth defect at low l-arabinose level [42].

### Negative influence of MtLat-1 on the induction of hemicellulase in *M. thermophila*

**Fig. 2:**
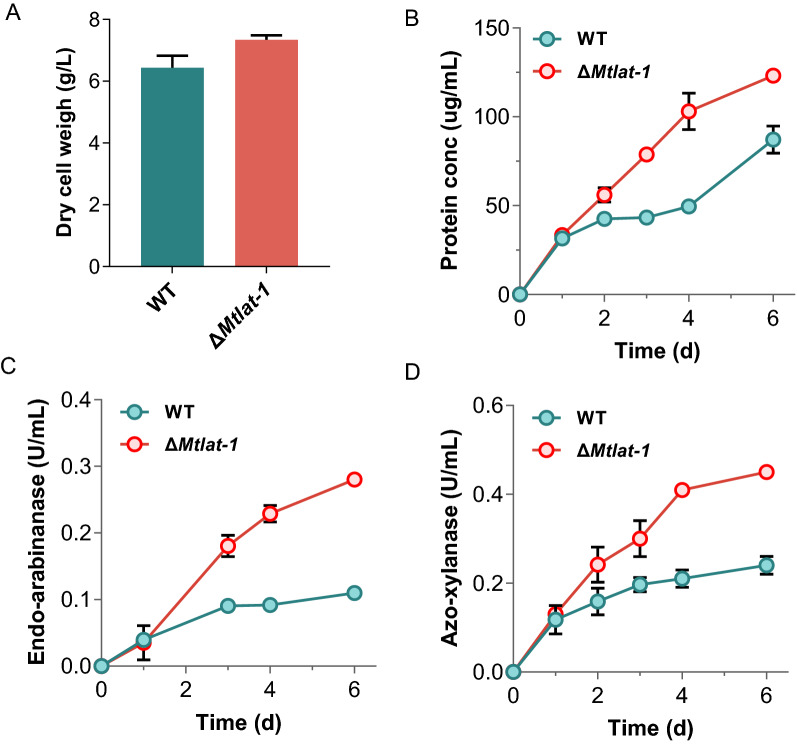
Growth phenotypes of the *M. thermophila* Δ*Mtlat-1* under arabinan condition. **A** Cell dry weight of *M. thermophila* wild-type (WT) and Δ*Mtlat-1* strains after growth in 1 × VMM with 2% arabinan for 4 days. **B** Protein concentrations, **C** arabinanase activity, and **D** xylanase activity of the culture supernatants for the *M. thermophila* strains grown in 2% arabinan medium. Error bars indicate the SD from at least three biological replicates

In lignocellulolytic fungi, pentose (d-xylose and l-arabinose) can act as inducers of the expression of genes encoding plant biomass-degrading enzymes [39, 43]. We hypothesized that the MtLat-1 protein, showing l-arabinose-specific transport activity, participates in sensing and transducing an l-arabinose signal that is involved in the induction of hemicellulolytic enzymes in *M. thermophila*. To test this hypothesis, physiological phenotypes of the strain Δ*Mtlat-1* were assayed when it was grown on arabinan or xylan. Unexpectedly, the strain Δ*Mtlat-1* exhibited an approximately 14% increase in dry cell weight compared with the WT when grown on arabinan for 4 days (Fig. 2a), which was different from *N. crassa,* where *lat-1* deletion attenuated the growth on arabinan [41]. In addition, compared with the WT strain, the secreted protein, arabinanase and xylanase activities of the *Mtlat-1* mutant increased by 108%, 149%, and 85%, respectively (Fig. 2b–d), indicating that the hemicellulase expression machinery was stimulated by disruption of *Mtlat-1*. However, when grown on xylan, the *Mtlat-1* mutant was similar to the WT in the phenotypes, including biomass accumulation, protein production and hemicellulolytic activity (Additional file 2: Fig. S2), consistent with the specific induction of *Mtlat-1* by l-arabinose.

To confirm that these phenotypes for growth on arabinan were attributable to the disruption of *Mtlat-1* rather than some other unknown genetic mutations, we complemented the mutant Δ*Mtlat-1* mutant with an ectopic copy of *Mtlat-1* under the control of the native promoter. The complementation strain Pn-Mtlat-1 exhibited completely restored l-arabinose uptake activity and showed similar phenotypes to the WT strain in protein secretion, and arabinanase and xylanase activities, when grown on arabinan (Additional file 2: Fig. S3). These results indicated that the l-arabinose transporter MtLat-1 negatively affects the production of hemicellulolytic enzymes when *M. thermophila* grown on arabinan.

### Transcriptomic analysis of the *Mtlat-1* deletion mutant on arabinan

**Fig. 3:**
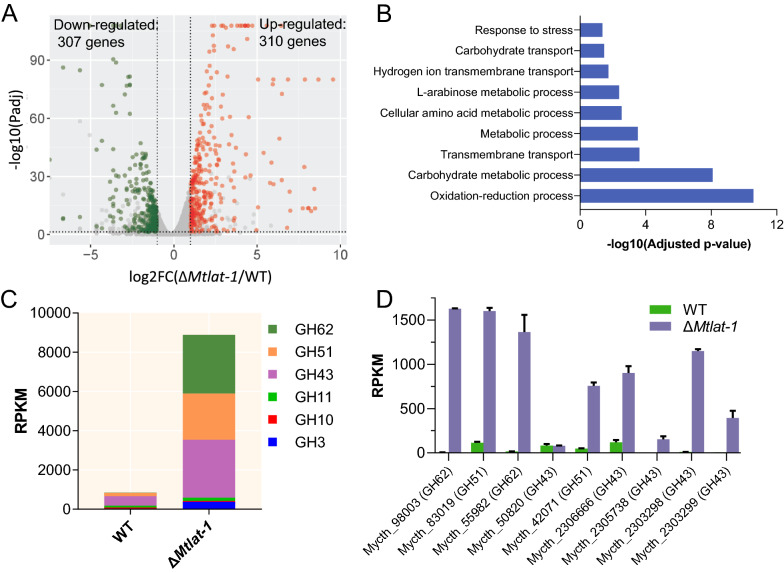
Comparative transcriptomic analysis of the *M. thermophila* strains WT and Δ*Mtlat-1* grown in 1 × VMM with 2% arabinan for 4 days. **A** Differential expression analysis of Δ*Mtlat-1* in comparison with the WT strain. Genes exhibiting significant differential expression levels are plotted in red (upregulated in strain Δ*Mtlat-1*) or green (downregulated in strain Δ*Mtlat-1*). **B** Enrichment of the genes with up-regulated expression in strain Δ*Mtlat-1* within the terms of Biological Process (BP) category during GO analysis. **C** Total expression of genes encoding major hemicellulases from RNA-Seq data. **D** Transcriptional profiles of genes encoding arabinanolytic enzymes in the Δ*Mtlat-1* and WT strains when grown in 1 × VMM with 2% arabinan for 4 days

To further investigate the effect of MtLat-1 on hemicellulase production, comparative transcriptomic profiles of the WT and Δ*Mtlat-1* strains grown in arabinan medium for 2 and 4 days, were determined using RNA-Seq analysis (Additional file 1: Table S2). Analysis of differential gene expression revealed that 617 genes were differentially expressed between the WT and Δ*Mtlat-1* strains after 4 days, of which 307 genes were significantly downregulated and 310 genes were markedly upregulated in strain Δ*Mtlat-1* at 4 days (Fig. 3a and Additional file 1: Table S4). Gene Ontology (GO) analysis of the up-regulated genes indicated that carbohydrate metabolic process, transmembrane transport, cellular amino acid metabolic process, l-arabinose metabolic process, carbohydrate transport and so on, were significantly enriched functional categories (Fig. 3b and Additional file 1: Table S6). Consistent with the rapid growth of strain Δ*Mtlat-1*, seven putative sugar transporter genes (Mycth_2305795, Mycth_43941, Mycth_96047, Mycth_104673, Mycth_2299846, Mycth_38796, Mycth_54010) exhibited markedly up-regulated expression levels in Δ*Mtlat-1* compared with the WT, which is a prerequisite for efficient sugar utilization and mycelium growth. An analysis of the transcriptional profiles of Carbohydrate-Active enzymes (CAZyme) genes (GH62, GH51, GH43, GH11, GH10, and GH3) indicated that genes encoding hemicellulolytic enzymes were dramatically induced in strain Δ*Mtlat-1* relative to the WT (Fig. 3c, Additional file 2: Fig. S4A and Additional file 1: Table S5). Eight of nine genes encoding arabinanolytic enzymes exhibited significantly up-regulated expression levels, of which four (Mycth_98003, arabinofuranosidase; Mycth_83019, arabinofuranosidase; Mycth_55982, arabinofuranosidase; and Mycth_2303298, arabinanase) with RPKM values > 1000 were up-regulated by more than tenfold in strain Δ*Mtlat-1* compared with the WT when growth on arabinan for 4 days (Fig. 2b). In plant biomass, arabinan is the component of side-chains of pectin and hemicellulose [44]. We also observed that four pectinolytic enzyme genes (Mycth_90594, *pyl-1*; Mycth_51766, *pyl-2*; Mycth_55717, *pyl-3*; and Mycth_55717, *gh28-1*) and three xylanolytic enzyme genes (Mycth_2301869, *gh43-5*; Mycth_104628, *gh3-7*; Mycth_89603, *gh11-1*) showed significantly increased expression levels in the Δ*Mtlat-1* mutant compared with the WT. In addition, the genes associated with the pentose catabolism were significantly up-regulated in Δ*Mtlat-1*, including xylose reductase (Xr, Mycth_43671), l-arabinitol 4-dehydrogenase (Ard, Mycth_62052), xylitol dehydrogenase (Xdh, Mycth_2293953), l-xylulose reductase (Lxr, Mycth_2302811), and d-xylulose kinase (Xks, Mycth_67060), which might attributed to increased l-arabinose release resulting from the enhancement of hemicellulolytic enzyme activity in Δ*Mtlat-1*.

### A non-transporting MtLat-1 mutant also participates in the induction of arabinanolytic enzymes

**Fig. 4:**
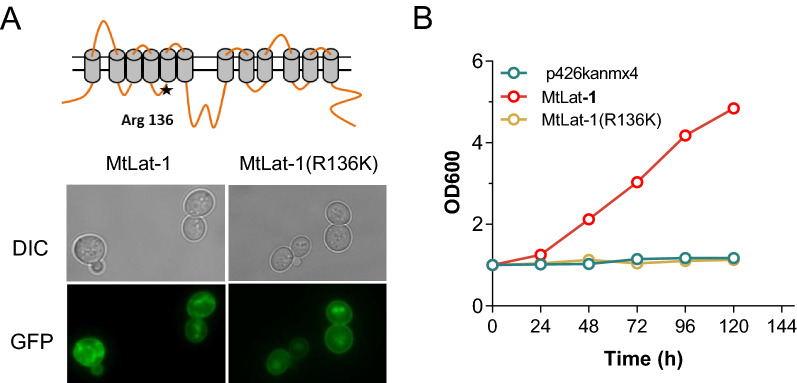
Non-transporting MtLat-1 point mutant. **A** Confocal fluorescence imaging of recombinant *Saccharomyces cerevisiae* BSW5AP strains expressing MtLAT-1 and point mutant MtLat-1(R136K), demonstrating correct localization to the yeast plasma membrane. A structural model of MtLat-1 was predicted based on TMHMM Server v. 2.0. **B** l-Arabinose-mediated growth of *S. cerevisiae* BSW5AP expressing MtLAT-1 or MtLat-1(R136K). Cells harboring the empty vector p426kanmx4 served as a negative control (CK). Error bars indicate the SD from at least three biological replicates

**Fig. 5:**
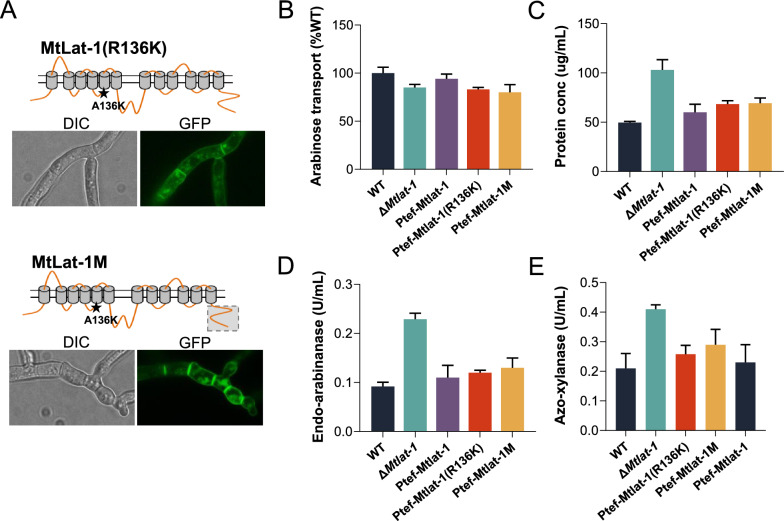
Growth phenotypes of the *M. thermophila* strains overexpressing mutant transporter MtLat-1(R136K) or MtLat-1M. **A** Subcellular localization of MtLat-1(R136K) and MtLat-1T in *M. thermophila*. **B** l-Arabinose transport rates of mycelia from the *M. thermophila* strains. Strain Ptef-Mtlat-1, overexpressing Mtlat-1 under the control of *tef-1* promoter in the Δ*Mtlat-1* mutant; Strain Ptef-Mtlat-1(R136K), overexpressing Mtlat-1(R136K) in the Δ*Mtlat-1* mutant; strain Ptef-Mtlat-1M, overexpressing Mtlat-1T in the Δ*Mtlat-1* mutant. **C** Protein concentrations, **D** Arabinanase activity, and **E** xylanase of the culture supernatants for the *M. thermophila* strains grown in 2% arabinan medium. Error bars indicate the SD from at least three biological replicates

In yeast species and some filamentous fungi, mutation to lysine of a conserved arginine residue (R to K) located in a cytoplasmic loop just preceding the fifth transmembrane domain of several hexose sensors, leads to the loss of substrate uptake activity, but constitutive glucose signaling [6, 11, 45, 46]. To determine whether MtLat-1 repressed the induction of arabinanolytic enzymes when the corresponding residue was altered, a mutant form of the protein, MtLat-1(R136K), was constructed. *S. cerevisiae* strain BSW5AP with a heterogenous arabinose metabolic pathway and native l-arabinose transporter gene deleted [47] was used for assay of substrate uptake. MtLat-1(R136K) fused to green-fluorescent protein (GFP) displayed correct plasma membrane localization of MtLat-1(R136K) in the recombinant BSW5AP strain (Fig. 4a). As expected, MtLat-1(R136K) could not support the growth of strain BSW5AP on l-arabinose, showing that the mutation abolished the transport function of MtLat-1 (Fig. 4b). When the mutant protein fused with GFP protein was introduced into the WT strain of *M. thermophila*, a GFP signal was detected in the cellular membrane of the fungal hyphae (Fig. 5a). Subsequently, the genes encoding MtLat-1 and MtLat-1(R136K) were separately overexpressed in the Δ*Mtlat-1* mutant under the control of the constitutive *tef-1* promoter to generate the resultant strains Ptef-Mtlat-1 and Ptef-Mtlat-1(R136K), respectively. The initial l-arabinose uptake rate of strain Ptef-Mtlat-1(R136K) was lower than that of mutant Ptef-Mtlat-1 (Fig. 5b), but the two mutants displayed a similar final cell yield when grown on arabinan (Additional file 2: Fig. S5). Consistently, the secreted protein content and hemicellulolytic activities of the strain Ptef-Mtlat-1(R136K) reached the similar levels to those of mutant Ptef-Mtlat-1 (Fig. 5c–e). These data indicated that hemicellulase gene expression was correlated with the signaling function of MtLat-1, but not its transport activity. The results supported the hypothesis that MtLat-1 protein senses and transduces the l-arabinose signal, which controls the downstream components of the hemicellulase induction pathway.

Previous studies have shown that C-terminal cytoplasmic sequence extension of several transceptors related to the CCR trigger or the induction of cellulase was required for their interaction with a component of the molecular signal transduction pathway [11, 48]. To investigate the effects of deletion of the cytoplasmic C-terminal tail of MtLat-1 on the induction of hemicellulase, a truncation mutant was constructed by deleting the C-terminal extension peptide (63 amino acids) of MtLat-1M, and this variant was introduced into the Δ*Mtlat-1* strain. When grown on arabinan, the resultant strain Ptef-Mtlat-1M showed mycelium yield, secreted protein content, and hemicellulolytic activities similar to those of the strain Ptef-Mtlat-1(R136K), revealing that the C-terminal cytosolic region of MtLat-1 was dispensable for the function of the protein in transmitting the l-arabinose signal.

### The expression of *Mtlat-1* is directly regulated by transcription factor MtAra-1

**Fig. 6:**
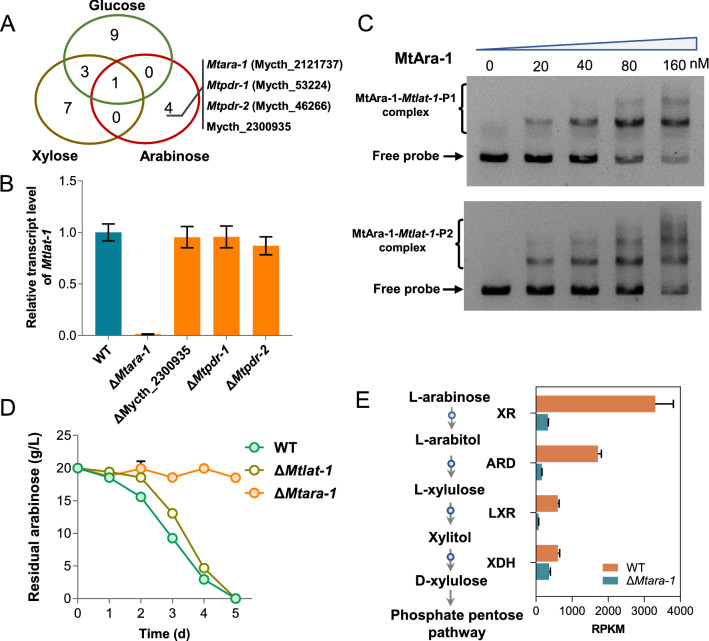
Transcription factor MtAra-1 directly regulates expression of *Mtlat-1*. **A** Venn diagram analysis of transcription factor genes with significantly upregulated expression levels in WT *M. thermophila* grown on l-arabinose, d-xylose, or d-glucose, compared with growth in no carbon conditions. **B** Relative expression levels of *Mtlat-1* in the mutants Δ*Mtara-1*, Δ*Mtpdr-1*, Δ*Mtpdr-2*, and ΔMycth_2300935, compared with the WT after the induction by l-arabinose for 4 h. Error bars indicate the SD from at least three biological replicates. **C** Electrophoretic mobility shift assays of the binding of MtAra-1 to the upstream region of *Mtlat-1*. Each lane contained 10 ng probe and the indicated amount of the purified DNA-binding domain (nM) of MtAra-1. **D** Sugar consumption by strain Δ*Mtara-1* when grown in 1 × VMM with 2% l-arabinose. **E** Expression levels of genes involved in l-arabinose catabolism in the Δ*Mtara-1* mutant of *M. thermophila* when grown on l-arabinose for 4 h. XR, xylose reductase (Mycth_43671); ARD, l-arabinitol 4-dehydrogenase (Mycth_62052); LXR, l-xylulose reductase (Mycth_2302811); XKS, d-xylulose kinase (Mycth_67060)

To search for transcription factors essential for expression of *Mtlat-1*, we determined transcriptomic profiles of all transcription factor genes in *M. thermophila* when grown on mono-saccharides (d-glucose, d-xylose, or l-arabinose) or under starvation (no carbon) conditions. As shown in Fig. 6a, a total of 24 genes were significantly induced by monosaccharides, compared with starvation condition. Given that a high expression level of *Mtlat-1* was specific to the presence of l-arabinose, we considered the four genes (Mycth_2121737; *MtPdr-1*, Mycth_53224; *MtPdr-2*, Mycth_46266; and Mycth_2300935) that were only highly induced by l-arabinose as the candidate regulators of *Mtlat-1.* Next, single-gene mutants of these genes were constructed and the expression level of *Mtlat-1* in response to l-arabinose was detected by real-time quantitative PCR (RT-qPCR) assay. Three of the mutants showed no significant change in *Mtlat-1* expression compared with the WT strain. However, the ΔMycth_2121737 strain showed dramatically decreased expression of *Mtlat-1* (Fig. 6b). Mycth_2121737 is a zinc binuclear cluster [Zn(II)_2_Cys_6_] DNA-binding protein and its orthologs in *N. crassa* (Ara-1) and *T. reesei* (Ara1) were related to utilization of arabinan, l-arabinose, and d-galactose [14, 27]. Therefore, in this study, Mycth_2121737 was named as MtAra-1.

To investigate whether MtAra-1 directly regulates *Mtlat-1* expression, we conducted electromobility shift assays (EMSAs), involving the DNA-binding domain (DBD) of MtAra-1 and the promoter region of *Mtlat-1*. A glutathione *S*-transferase (GST)-fused MtAra-1-DBD was expressed in and purified from *Escherichia coli*. Two probe fragments of the promoter region of *Mtlat-1* (nucleotide positions − 396 to − 1 and − 860 to − 396, respectively) were amplified by PCR. In the EMSAs, the recombinant MtAra-1 bound the *Mtlat-1* promoter regions in a typical protein concentration-dependent manner. Retardation occurred upon addition of 10 nM recombinant MtAra-1 protein (Fig. 6c).

### Transcription factor MtAra-1 substantially affects l-arabinose release and catabolism

The deletion of *Mtara-1* abolished growth of *M. thermophila* on l-arabinose (Fig. 6d), indicating that MtAra-1 was not related only to the expression of *Mtlat-1*. A comparative transcriptomic analysis revealed that the genes involved in pentose catabolism, including *xr*, *lad*, *xdh*, *lxr*, and *xks*, showed a significant decrease in expression levels in the Δ*Mtara-1* mutant compared with those in the WT strain when grown on l-arabinose (Fig. 6e). Six putative pentose transporter genes (*Mtlat-1*, Mycth_2305795, Mycth_96047, Mycth_2302949, Mycth_112491, and Mycth_108924) were also dramatically downregulated in Δ*Mtara-1* grown on l-arabinose (Additional file 2: Fig. S6). However, strain Δ*Mtara-1* grew similar to the WT on xylose (Additional file 2: Fig. S1B), indicating that the expression of the genes *xr*, *xdh*, and *xks*, which are shared by l-arabinose and d-xylose metabolism, was induced independently of MtAra-1 in the presence of xylose (Additional file 1: Table S2). In *M. thermophila*, Xlr1 is the main regulator of the expression of xylanolytic genes and the genes involved in xylose utilization [25]. In filamentous fungi, Ard (l-arabinitol-4-dehydrogenase), also catalyzes the third reaction of the oxidoreductive catabolism of d-galactose. Consequently, we also found that the Δ*Mtara-1* strain showed a severely decreased rate of d-galactose consumption (Additional file 2: Fig. S2), consistent with observations in *N. crassa* and *T. reesei* [14, 27]. Unexpectedly, growth phenotype analysis indicated that *Mtara-1* disruption significantly increased the d-glucose consumption rate of *M. thermophila*, indicating a complex regulatory network of d-glucose and pentose metabolism.

**Fig. 7:**
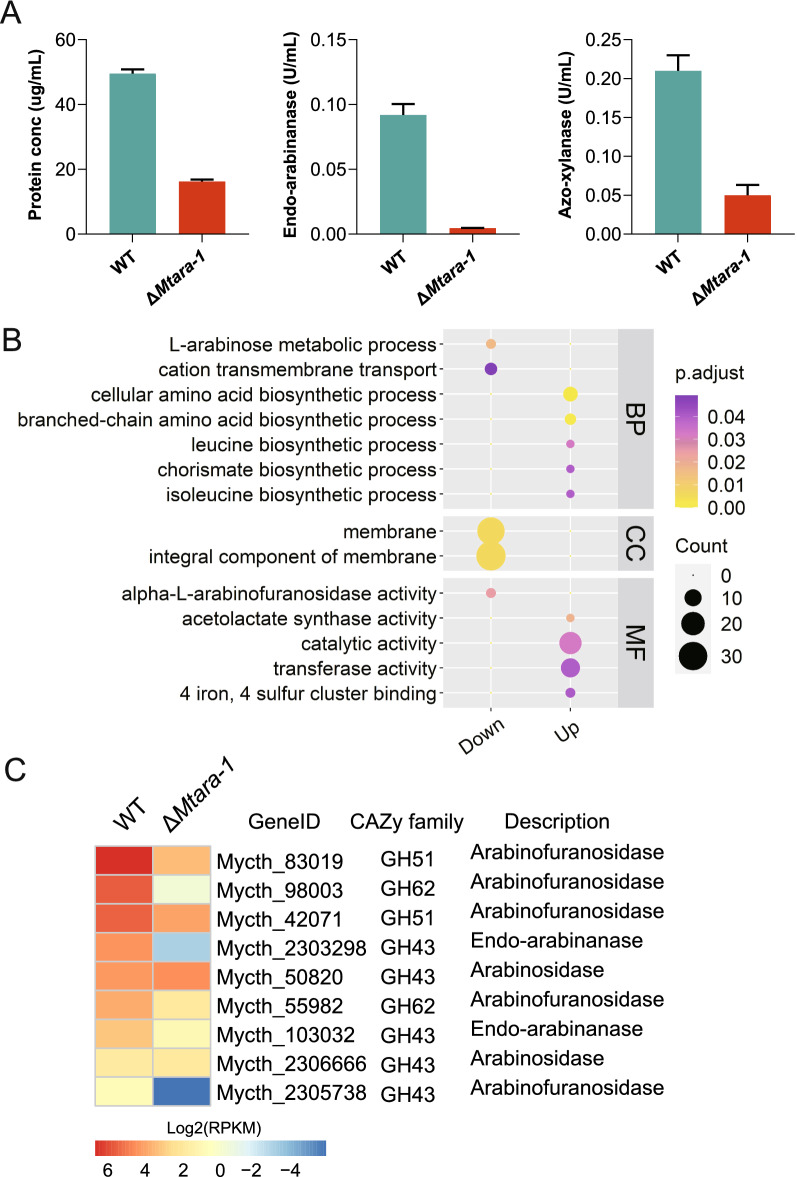
Essential role of MtAra-1 in arabinan degradation and utilization. **A** Protein concentrations, arabinanase activity, and xylanase activity of the culture supernatants of *M. thermophila* strain Δ*Mtara-1* grown in 2% arabinan medium for 4 d. **B** Gene ontology analysis of the genes showing downregulated expression levels in strain Δ*Mtara-1* compared with the WT after the induction with arabinan for 4 h. Down, the genes with down-regulated expression levels in Δ*Mtara-1*; Up, the genes with up-regulated expression levels in Δ*Mtara-1*; BP, Biological Process; CC, Cellular Component; MF, Molecular Function. **C** Heatmap analysis of expression levels of genes encoding arabinanolytic enzymes in the Δ*Mtara-1* and WT strains under arabinan condition. Log-transformed expression values are color-coded

To further dissect the effects of the deletion, we compared protein secretion and the hemicellulolytic/cellulolytic activities of strains WT and Δ*Mtara-1* when grown on plant biomass. As shown in Fig. 7a, the Δ*Mtara-1* mutant showed a dramatical reduction in total secreted protein production, xylanase activity and arabinase activity compared with the WT strain when grown on arabinan. Similar results were also obtained with xylan (Additional file 2: Fig. S7A). However, only a slight effect on protein production and cellulase activity of strainΔ*Mtara-1* was observed for growth on Avicel (cellulose microcrystal) (Additional file 2: Fig. S7B). Next, to further investigate the role of MtAra-1 in arabinan utilization, we transcriptionally profiled the response of the WT and Δ*Mtara-1* strains to arabinan induction for 4 h. In the presence of arabinan, the expression levels of 308 genes were significantly downregulated inΔ*Mtara-1* compared with the WT (Additional file 1: Table S8). GO enrichment analysis revealed that the downregulated genes in the Δ*Mtara-1* mutant were mainly involved in l-arabinose metabolism, amino acid biosynthetic process, transmembrane transport, and arabinofuranosidase activity (Fig. 7b). When transcription profiles of CAZyme genes were analyzed, we found that all the genes encoding arabinanolytic enzymes exhibited markedly decreased expression levels in strain Δ*Mtara-1* compared with the WT (Fig. 7c), showing their high dependence on MtAra-1. However, only one gene involved in xylan degradation (Mycth_52904) was included among the downregulated genes, suggesting that MtAra-1 is mainly involved in inducing expression of arabinanolytic enzyme genes. The reduced secreted protein production and xylanase activity in the Δ*Mtara-1* mutant when grown on xylan may be attributable to the low purity of the xylan used.

## Discussion

Filamentous fungi are able to secrete a series of lignocellulosic enzymes to degrade the plant biomass to fermentable sugars, and have been exploited as the main source of commercial hydrolases for biotechnological applications [15]. During the degradation of lignocellulose, the released mono/oligo-saccharides enter the cytoplasm via membrane sugar transporters to provide energy and building blocks, or serve as molecular signals that regulate hydrolytic enzyme production. Several membrane proteins that serve as molecular sensors and sugar transporters play important roles in nutrient acquisition and the induction of lignocellulolytic enzyme genes, such as Hgt-1/2, CDT-1/2 and CLP1 from *N. crassa*, and Crt1 and Stp1 from *T. reesei* [6–9].

Our previous study revealed that sugar transporter MtLat-1 of *M. thermophila* displays a high uptake activity and specificity for l-arabinose [38]. LAT-1 is conserved among ascomycete fungi. The closest relatives of LAT-1 that have been biochemically characterized include Lat-1 (Trire2_104072) from *T. reesei*, NcLat-1 (NCU02188) from *N. crassa*, LAT-1 from *Ambrosiozyma monospora* [49], and AraT from *Pichia stipitis* [50] and *Penicillium chrysogenum* [51]. Although all these proteins function as l-arabinose transporters, their effects on physiological phenotypes differ in various fungi when grown on l-arabinose and arabinan. In *T. reesei*, Lat-1 is a high-affinity symporter and shows high specificity for l-arabinose. The absence of Trire2_104072 did not result in an altered growth phenotype or different total protein secretion when grown on in high (1%) concentration of l-arabinose, lactose, or spent grain extract with a high content of arabinoxylan [42]. However, a severe growth defective of an *N. crassa lat-1* mutant was observed on arabinan. Moreover, in *N. crassa*, *lat-1* disruption caused a remarkably reduced l-arabinose uptake, which abolished the induction of *ard-1* (gene encoding l-arabinitol-4-dehydrogenase) at low (2 μM) l-arabinose concentrations [41]. Herein, the expression of *Mtlat-1* was specifically induced by l-arabinose and its absence resulted in a decrease in l-arabinose uptake and consumption rates, but not those of d-glucose, d-xylose, or d-galactose, which is consistent with the specific l-arabinose transport activity of MtLat-1 [38]. However, mycelium growth, secreted protein production, and hemicellulolytic enzyme activities were markedly pronounced in the Δ*Mtlat-1* mutant when grown on arabinan. Comparative transcriptomic analysis revealed that deletion of *Mtlat-1* enhanced the expression of genes encoding xylanolytic enzymes and arabinanolytic enzymes. These results suggested that l-arabinose transporter MtLat-1 repressed the expression of hemicellulolytic enzymes on arabinan but not on xylan. Furthermore, a point mutant of MtLat-1 in which the transport activity was abolished exhibited similar effects to the wild-type MtLat-1 on the protein secretion and hemicellulolytic activities on arabinan, clearly demonstrating that the involvement of MtLat-1 in *M. thermophila* hemicellulase gene expression was independent of its transport activity. These experiments have helped to separate nutrient sensing from substrate transport, and indicate that MtLat-1 protein serves as a transceptor, which might sense and transduce molecular signal of l-arabinose, one of the end products of hemicellulose degradation, to repress the induction of hemicellulase genes. The role of transceptor in the induction of genes involved in plant biomass degradation has also been demonstrated for CDT-1/2 in *N. crassa* and Crt1 in *T. reesei* [9, 52].

The C-terminal cytoplasmic fragments of several transporters and sensors have been reported to be essential for signal transduction [11]. Truncation of some *C*-terminal amino acids did not alter their transport activities, but abolished their signaling function [48, 52]. In *T. reesei*, a Crt1 mutant retaining only the first five amino acids of C-terminus still had the lactose transport activity but lost the cellulase induction activity [52]. In *M. thermophila*, MtLat-1 has a C-terminal extension of 63 amino acids, which is markedly shorter than those of Snf3 (303 amino acids) and Rgt2 (220 amino acids), but longer than that of Crt1 (44 amino acids) [11, 52]. However, an MtLat-1 mutant with truncation of the entire C*-*terminus restored the growth phenotype and hemicellulolytic activities of strain Δ*Mtlat-1* to similar levels to those of the WT on arabinan, showing that the C-terminal cytosolic region of MtLat-1 is not required for its signaling function. Similarly, in *Pichia pastoris*, deletion of the C-terminal fragment (150 amino acids) of glucose sensor Gss1 slightly affected glucose catabolite repression and pexophagy, but the signaling function of the protein was maintained [53]. Signal transduction by transporters and sensors can be mediated by their relocation from the cell membrane to intracellular compartments, including endosomes, the Golgi apparatus, and nuclear membranes [54]. In *T. reesei*, the internalization of Crt1 was observed on cellulose, although it was also found under glucose or glycerol and may not be correlated with the induction of cellulases [52]. Herein, MtLat-1 was mainly localized in the cellular membrane and dual localization of MtLat-1–GFP was not observed. The precise mechanism by which MtLat-1 mediates the signal transduction involved in hemicellulase induction requires further investigation.

Transcription factors play an essential role in regulation of the expression of CAZyme genes and genes involved in sugar transport and intracellular catabolism. Several essential transcription factors associated with hemicellulose deconstruction and utilization have been studied in fungi, including CreA/CRE-1, Xyr1/XlnR, and Ara-1. CreA/CRE-1 is a major regulator of CCR, a process through which the expression of genes involved in the utilization of non-preferred carbon sources, including hemicellulose/cellulose, are inhibited [18, 55]. In *T. reesei*, XlnR/XYR1 regulates both cellulase and hemicellulase genes, while it is a major positive activator of hemicellulase genes in *N. crassa* and *F. graminearum* [16, 21, 24]. Similarly, in *M. thermophila*, Xlr1 controls the expression of xylanolytic genes and genes involved in pentose transport and catabolism, but it has a smaller impact on l-arabinose catabolism [25]. In several fungi, Ara-1 is essential for expression of d-galactose and l-arabinose releasing and catabolic genes. In *N. crassa* and *T. reesei*, deletion of *ara-1* dramatically downregulated the expression of l-arabinose transporter Lat-1 [27, 42]. In *M. thermophila*, the induced expression of *Mtara-1* was observable only on l-arabinose, as also observed for *Mtlat-1*. Transcriptional analysis and EMSAs demonstrated that MtAra-1 directly regulates *Mtlat-1* expression by binding to its promoter region. Consistent with observations in *N. crassa* [14, 27],

*Mtara-1* disruption abolished the growth of *M. thermophila* on l-arabinose and d-galactose, but no effect on the growth of strain Δ*Mtara-1* was observed on d-xylose. Moreover, the Δ*Mtara-1* mutant exhibited a remarkable reduction in secreted protein production and hemicellulolytic activities when grown on arabinan. Our transcriptomic data reflected the significantly downregulated expression of all arabinanolytic enzyme-encoding genes in the Δ*Mtara-1* mutant when it was grown on arabinan. These results demonstrated that MtAra-1 is mainly involved in regulation of the expression of arabinanolytic genes and the genes associated with l-arabinose and d-galactose catabolism in *M. thermophila*.

## Conclusion

In this study, we demonstrated that MtLat-1 is involved in l-arabinose transport and signal transduction associated with the suppression of expression of hemicellulolytic enzyme-encoding genes in *M. thermophila*. The absence of *Mtlat-1* caused a decrease on l-arabinose uptake and consumption rates, but led to increases in mycelium growth, secreted protein production, and hemicellulolytic enzyme activities on arabinan. Furthermore, point mutation of MtLat-1 revealed that the involvement of MtLat-1 in the expression of hemicellulase genes is independent of its transport activity. Moreover, transcription factor MtAra-1 played an important role in the induction of arabinanolytic genes and l-arabinose catabolism, and directly regulated *Mtlat-1* expression by binding to its promoter region.

## Materials and methods

### Strains and growth conditions

*M. thermophila* ATCC 42464 was obtained from the American Type Culture Collection (ATCC). This WT strain and its mutants were grown on Vogel’s minimal medium (VMM) with 2% (w/v) glucose at 35 °C to obtain mature conidia. Antibiotic was added when needed to screen for transformants. For media shift experiments, *M. thermophila* strains were precultured in 100 mL of 1 × VMM with 2% glucose for 16 h. Subsequently, the mycelia were collected, washed three times with 1 × VMM, and then transferred to 100 mL of fresh 1 × VMM with 2% l-arabinose (Sigma) or arabinan (Megazyme) for continued incubation for 2 h. For fungal growth assays, mature conidia were inoculated into 100 mL of 1 × VMM with 2% carbon source (glucose, d-xylose, l-arabinose, arabinan, xylan, or Avicel) at a concentration of 2.5 × 10^5^ conidia/mL in 250-mL Erlenmeyer flasks. The culture was incubated at 45 °C with rotary shaking at 150 rpm.

*S. cerevisiae* BSW5AP with heterogenous arabinose metabolic pathway [47], was cultured in YPD medium (10 g/L yeast extract, 20 g/L peptone, and 20 g/L glucose) at 30 °C. The medium was supplemented with 400 g/mL Geneticin (G418; Amresco) when required to maintain a plasmid with the KanMX4 marker. For assay of growth properties, a single colony from a fresh agar plate was precultured in 10 mL of YPD medium supplemented with 400 g/mL G418. Cells in the late exponential phase were washed with sterile water, and then inoculated into 30 mL 2% l-arabinose medium in 250-mL Erlenmeyer flasks at an initial OD_600_ value of 1.0. Cells were cultivated in an orbital shaker at 200 rpm at 30 °C and samples were taken at intervals.

*E. coli* Mach-T1 cells were used for vector manipulation and propagation, and were cultivated in Luria–Bertani medium with 100 µg/mL ampicillin or 50 µg/mL kanamycin for plasmid selection.

### Plasmid and strain construction

The primer sequences used in this study are listed in Additional file 1: Table S1. For the deletion of target genes using the CRISPR/Cas9 system, the guide RNA (gRNA) expression cassettes were constructed as described previously [40]. Specific sgRNA target sites in target genes (*Mtlat-1*, Mycth_95427; *Mtara-1*, Mycth_2121737; *MtPdr-1*, Mycth_53224; *MtPdr-2*, Mycth_46266; Mycth_2300935) were identified using the sgRNACas9 tool [56]. Fragments, including the *M. thermophila* U6 promoter sequence, the synthetic gRNA scaffold sequence, and the target DNA sequence were constructed via overlapping PCR and cloned into the pJET1.2/blunt cloning vector to generate the corresponding plasmids U6p-Mtlat-1-sgRNA, U6p-Mtara-1-sgRNA, U6p-MtPdr-1-sgRNA, U6p-MtPdr-2-sgRNA, and U6p-Mycth_2300935-sgRNA. To construct donor DNA sequences, the 5′- and 3′-flanking fragments of *Mtlat-1*, *Mtara-1*, *MtPdr-1*, *MtPdr-2*, and Mycth_2300935 were separately amplified by PCR from *M. thermophila* genomic DNA, fused with the selectable marker cassette PtrpC-neo from plasmid p0380-neo [57], and cloned into pPK2BarGFP to generate donor DNA sequences Donor-Mtlat-1-neo, Donor-Mtara-1-neo, Donor-MtPdr-1-neo, Donor-MtPdr-2-1-neo, and Donor-Mycth_22300935-neo, respectively.

To generate the complementation strain, a DNA fragment containing the upstream region (1485 bp), the downstream region (853 bp), and full-length *Mtlat-1* (1934 bp), was amplified from the *M. thermophila* genome and ligated between the *Bgl*II and *Bam*HI sites of pAN52-PgpdA-bar [33] to generate the corresponding complementation plasmid pAN52-bar-Mtlat, using a NEB Gibson Assembly Kit. For overexpression of target genes, the genes encoding MtLat-1, MtLat-1(R136K), MtLat-1M, and their GFP fusion proteins were amplified by PCR and inserted into pAN52-Ptef1-TtrpC-bar [58] at the *Spe*I and *Bam*HI sites, under the control of the strong constitutive *tef1* (Mycth_2298136) promoter of *M. thermophila*, to generate the corresponding recombinant vectors. Polyethylene glycol (PEG)-mediated protoplast transformation for gene disruption or overexpression in *M. thermophila* was performed as described previously [40].

For expression of transporters in *S. cerevisiae* strain BSW5AP, the genes encoding MtLat-1, MtLat-1–GFP, MtLat-1(R136K), and MtLat-1(R136K)–GFP were separately inserted into the shuttle plasmid p426kanmx4 [38] with the selective marker KanMX4, behind the PGK promoter. *S. cerevisiae* transformations were carried out as described previously [59].

### GFP fluorescence and confocal fluorescence microscopy

To detect subcellular localization of MtLat-1 and its mutants in *M. thermophila*, mycelia were pre-grown in VMM with 2% arabinose for 16 h at 45 °C and then the cells were observed using the Olympus BX51 fluorescence microscopy system. Recombinant *S. cerevisiae* BSW5AP strains expressing sugar transporters tagged with GFP were inoculated into YPD medium and grown to the exponential phase (OD_600_ ~ 1.0). The cells were collected, washed twice with sterile water, and then resuspended in sterile water. Next, 10 μL of culture was spotted on a cover glass for confocal microscopy. The images were processed using ImageJ software.

### Sugar uptake assays of *M. thermophila* strains

The *M. thermophila* strains were precultured in 1 × VMM containing 2% glucose for 18 h. Then, mycelia were washed three times in 1 × Vogel’s salts without any carbon source and shifted into 0.5% arabinose medium for the additional 4 h of induction. After that, the mycelia were harvested, washed again as above, and resuspended in uptake buffer (1 × Vogel’s salts plus 10 mM arabinose and 10 μg/mL cycloheximide) for 20 min. The residual sugar in the supernatant was determined using HPLC with an e2695 instrument (Waters, Manchester, United Kingdom), and the fungal biomass was completely dried to determine the dry weight for data normalization.

### Protein and enzyme activity assays

The mature conidia of *M. thermophila* strain were inoculated into 100 mL of 1 × VMM with 2% carbon source (arabinan, xylan, or Avicel) at a concentration of 2.5 × 10^5^ conidia/mL in 250-mL Erlenmeyer flasks and incubated at 45 °C with rotary shaking at 150 rpm. Samples were taken at the indicated time for the assays of secreted protein and enzyme activity. The total extracellular protein in culture supernatants was measured using a Bio-Rad protein assay kit. Endo-1,5-l-arabinanase, endo-1,4-xylanase, and endo-glucanase activities in the supernatants of culture of *M. thermophila* strains were measured using an Azo-Xylan kit (Megazyme), an endo-1,5-l-arabinanase assay kit (Megazyme), and an Azo-CM-Cellulose assay kit (Megazyme), respectively.

### RNA extraction and transcriptional analysis

*M. thermophila* mycelia were shifted into 1 × VMM with 2% carbon source (glucose, xylose, arabinose, or arabinan) for the induction of 4 h, harvested by vacuum filtration, and immediately ground to a fine powder using a pestle and mortar with liquid nitrogen for the subsequent extraction of total RNA [55]. For transcriptome analysis of the *Mtlat-1* deletion mutant grown on arabinan, *M. thermophila* wild-type strain and *Mtlat-1* deletion mutant were cultured in 1 × VMM with 2% arabinan (Sampled at 2 days and 4 days).

RNA integrity and concentration were determined using agarose gel electrophoresis and NanoDrop spectrophotometer. Purified RNA samples, with RNA integrity number > 8.0, determined using an Agilent 2100 Bioanalyzer (Agilent Technologies), were sequenced by Novogene Corporation (Tianjin, China) using the Illumina NovaSeq 6000 platform to generate 150-bp paired-end reads.

Clean reads were mapped to the *M. thermophila* ATCC42446 genome sequence [32] using Tophat (v2.0.8b) [60]. The counts of reads mapped to unique exons were calculated by Htseq-count (V0.13.5) with SAM files and the GFF file as input and used for normalizing transcript abundance (RPKM, reads per kilobase per million mapped reads). DESeq package was used for analysis of differential gene expression [61]. Unless otherwise noted, genes with |log_2_ fold-change|≥ 1, RPKM ≥ 20 (at least one sample), and DESeq *P*-adj value < 0.05, were considered significantly differentially expressed between two samples. The detailed data are shown in Additional file 1: Table S2. The raw reads of transcriptomic data were deposited in the Gene Expression Omnibus (accession number: GSE221464) at the National Center for Biotechnology Information.

### Real-time quantitative PCR

For RT-qPCR analysis, precultured mycelia of *M. thermophila* strains were shifted into induction medium containing 2% arabinose as the carbon source and incubated for 4 h, then harvested for the subsequent extraction of total RNA using the method described previously [55]. Quantitative PCR was performed with SYBR Green Real-time PCR Master Mix (Toyobo, Osaka, Japan) using a CFX96 real-time PCR detection system (Bio-Rad). Negative controls contained an equal volume of water instead of RNA. The relative transcript level of each gene was calculated by the 2^−ΔΔCt^ method with the actin-encoding gene (Mycth_2314852) as the internal control.

### Electrophoretic mobility shift assays

The DNA sequence encoding the DBD of transcription factor MtAra-1 (residues 9–68, identified in NCBI) was amplified from *M. thermophila* cDNA and inserted between the *Bam*HI and *Xho*I sites of pGEX-4T-1 (GE Healthcare) to form a GST-tagged protein expression plasmid. The recombinant plasmid was introduced into *E. coli* BL21 (DE3) cells for protein expression and purification as previously described [37].

EMSAs were performed as described previously [37]. Briefly, promoter regions of *Mtlat-1* (P1, − 396 to − 1; P2, − 860 to − 396) were amplified from *M. thermophila* genomic DNA using primers shown in Additional file 1: Table S1. In each EMSA, recombinant MtAra-1 were incubated with a constant amount (10 ng) of the DNA probe at 25 °C for 30 min in buffer.

## Supplementary Information

**Additional file 1. Table S1**: List of PCR primers used in this study. **Table S2**: Profiles of RNA-Seq reads mapped to the genome of *M. thermophila*. **Table S3**: Transcriptomic profiles of 26 sugar transporters with robust expression levels (RPKM > 20) in at least one tested condition. **Table S4**: Genes showing significantly different transcriptional levels in strain *∆Mtlat-1* compared with the WT when grown on 1 × VMM with 2% arabinan for 4 days. **Table S5**: Transcriptomic profiles of genes encoding major hemicellulases from RNA-Seq data when grown on 1 × VMM with 2% arabinan. **Table S6**: Gene ontology (GO) analysis of up-regulated genes in strain *ΔMtlat-1* compared with the WT when grown on 1 × VMM with 2% arabinan for 4 days. **Table S7**: Transcriptomic profiles of transcription factor genes with significantly upregulated expression levels in WT *M. thermophila* grown on l-arabinose, d-xylose, or d-glucose, compared with that under no carbon. **Table S8**: Genes showing significantly different transcriptional levels in strain *∆Mtara-1* compared with the WT strain when grown on 2% arabinan for the induction of 4 h.

**Additional file 2. Fig. S1**: Sugar consumption by *M. thermophila* strains WT, *ΔMtlat-1*, and *ΔMtara-1*, when grown in 1 × VMM with 2% d-glucose (**A**), 2% d-xylose (**B**), or 2% d-galactose (**C**). Error bars indicate the SD from at least three biological replicates. **Fig. S2**: Growth phenotypes of the *M. thermophila* *ΔMtlat-1* mutant under xylan condition. **A** Cell dry weight of *M. thermophila* strains WT and *ΔMtlat-1* after growth on 2% xylan for 2 d. **B** Protein concentrations, **C** xylanase activity, and **D** arabinanase activity of the culture supernatants for the *M. thermophila* strains grown in 2% xylanase medium. Error bars indicate the SD from at least three biological replicates. **Fig. S3**: Growth phenotypes of the complementation strain of the *ΔMtlat-1* mutant. **A** l-arabinose transport rates of mycelia from the complementation strain Pn-Mtlat-1. **B** Cell dry weight of the *M. thermophila* complementation strain after growth on 2% arabinan for 4 days. **C** Protein concentrations, **D** arabinanase activity, and **E** xylanase activity of the culture supernatants for *M. thermophila* grown in 2% arabinan medium. Error bars indicate the SD from at least three biological replicates. **Fig. S4**: Comparative transcriptomic analysis of the WT and *ΔMtlat-1* *M. thermophila* strains grown in arabinan medium for 2 d. **A** Total expression of genes encoding major hemicellulases from RNA-Seq data. **B** Transcriptional profiles of genes encoding arabinanolytic enzymes in the *ΔMtlat-1* and WT strains when grown on 2% arabinan for 4 days. **Fig. S5**: Cell dry weight of *M. thermophila* strains after growth on 2% arabinan for 4 days. **Fig. S6**: Heatmap analysis of expression profiles for putative sugar transporter genes with statistically significant differences in transcript levels between *ΔMtara-1* and the WT under l-arabinose condition. Log-transformed expression values are color-coded. **Fig. S7**: Protein concentrations and hemicellulase/cellulase activity of the culture supernatants for *M. thermophila* strain *ΔMtara-1* grown in 1 × VMM with 2% xylan (**A**) or 2% Avicel (**B**) for 4 days.

## Data Availability

The data sets used and/or analyzed during the current study are available from the corresponding author on reasonable request.

## Abbreviations

Ard: l-Arabinitol-4-dehydrogenase
GFP: Green fluorescent protein
Lxr: l-Xylulose reductase
Transceptor: Transporter and acceptor
Xr: Xylose reductase
Xks: d-Xylulose kinase
Xdh: Xylitol dehydrogenase
WT: Wild-type
VMM: Vogel’s minimal medium
NoC: No carbon
Glu: d-Glucose
Xyl: d-Xylose
Ara: l-Arabinose
