# Supplementary material for: The arabinose transporter MtLat-1 is involved in hemicellulase repression as a pentose transceptor in Myceliophthora thermophila
Source: Biotechnol Biofuels Bioprod. 2023 Mar 25;16:51. doi: 10.1186/s13068-023-02305-3 (PMC10040116; doi:10.1186/s13068-023-02305-3)
Supplement: Supplementary file 2 — Additional file 2. Fig. S1: Sugar consumption by M. thermophila strains WT, ΔMtlat-1, and ΔMtara-1, when grown in 1 × VMM with 2% d-glucose (A), 2% d-xylose (B), or 2% d-galactose (C). Error bars indicate the SD from at least three biological replicates. Fig. S2: Growth phenotypes of the M. thermophila ΔMtlat-1 mutant under xylan condition. A Cell dry weight of M. thermophila strains WT and ΔMtlat-1 after growth on 2% xylan for 2 d. B Protein concentrations, C xylanase activity, and D arabinanase activity of the culture supernatants for the M. thermophila strains grown in 2% xylanase medium. Error bars indicate the SD from at least three biological replicates. Fig. S3: Growth phenotypes of the complementation strain of the ΔMtlat-1 mutant. A l-arabinose transport rates of mycelia from the complementation strain Pn-Mtlat-1. B Cell dry weight of the M. thermophila complementation strain after growth on 2% arabinan for 4 days. C Protein concentrations, D arabinanase activity, and E xylanase activity of the culture supernatants for M. thermophila grown in 2% arabinan medium. Error bars indicate the SD from at least three biological replicates. Fig. S4: Comparative transcriptomic analysis of the WT and ΔMtlat-1 M. thermophila strains grown in arabinan medium for 2 d. A Total expression of genes encoding major hemicellulases from RNA-Seq data. B Transcriptional profiles of genes encoding arabinanolytic enzymes in the ΔMtlat-1 and WT strains when grown on 2% arabinan for 4 days. Fig. S5: Cell dry weight of M. thermophila strains after growth on 2% arabinan for 4 days. Fig. S6: Heatmap analysis of expression profiles for putative sugar transporter genes with statistically significant differences in transcript levels between ΔMtara-1 and the WT under l-arabinose condition. Log-transformed expression values are color-coded. Fig. S7: Protein concentrations and hemicellulase/cellulase activity of the culture supernatants for M. thermophila strain ΔMtara-1 grown in 1 × VMM with [file 13068_2023_2305_MOESM2_ESM.docx]

**Additional file 2: Additional Figures**

**for**

**The arabinose transporter MtLat-1 is involved in hemicellulase induction as a pentose transceptor in *Myceliophthora thermophila***

Gu *et al.*


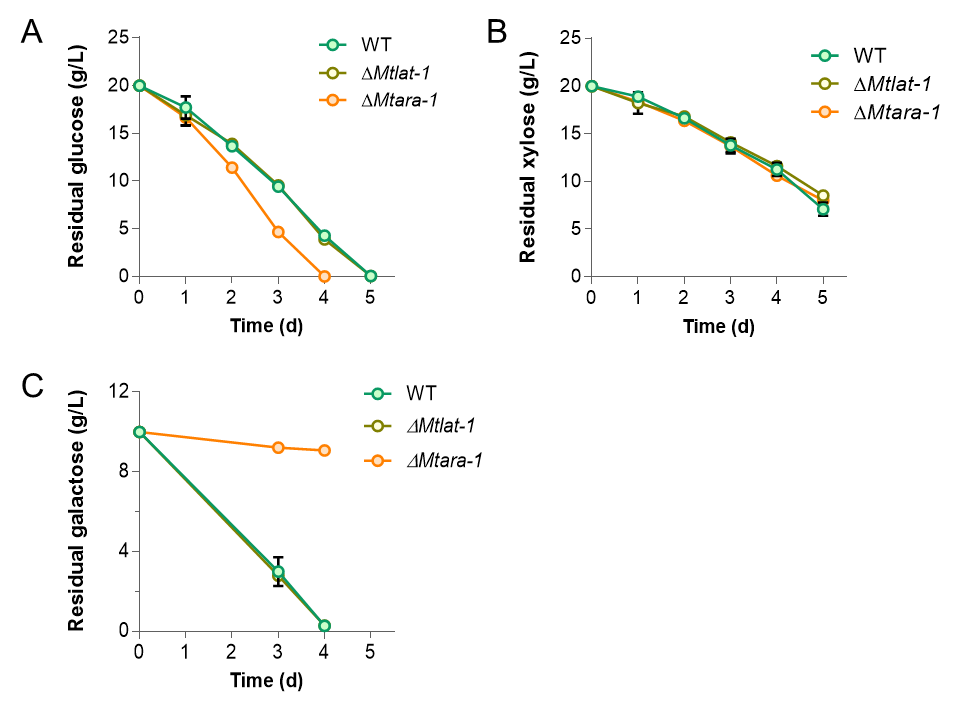


**Fig. S1** Sugar consumption by *M. thermophila* strains WT, Δ*Mtlat-1*, and Δ*Mtara-1*, when grown in 1 × VMM with 2% D-glucose (A), 2% D-xylose (B), or 2% D-galactose (C). Error bars indicate the SD from at least three biological replicates.


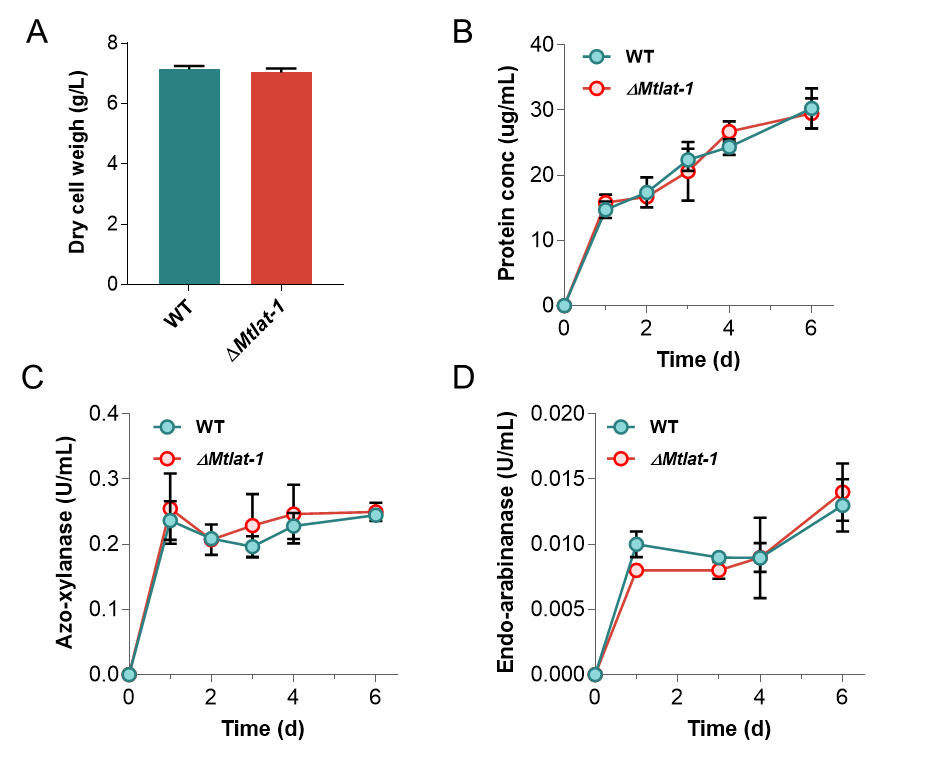


**Fig. S2** Growth phenotypes of the *M. thermophila* Δ*Mtlat-1* mutant under xylan condition. (A) Cell dry weight of *M. thermophila* strains WT and Δ*Mtlat-1* after growth on 2% xylan for 2 d. (B) Protein concentrations, (C) xylanase activity, and (D) arabinanase activity of the culture supernatants for the M. thermophila strains grown in 2% xylanase medium. Error bars indicate the SD from at least three biological replicates.

**
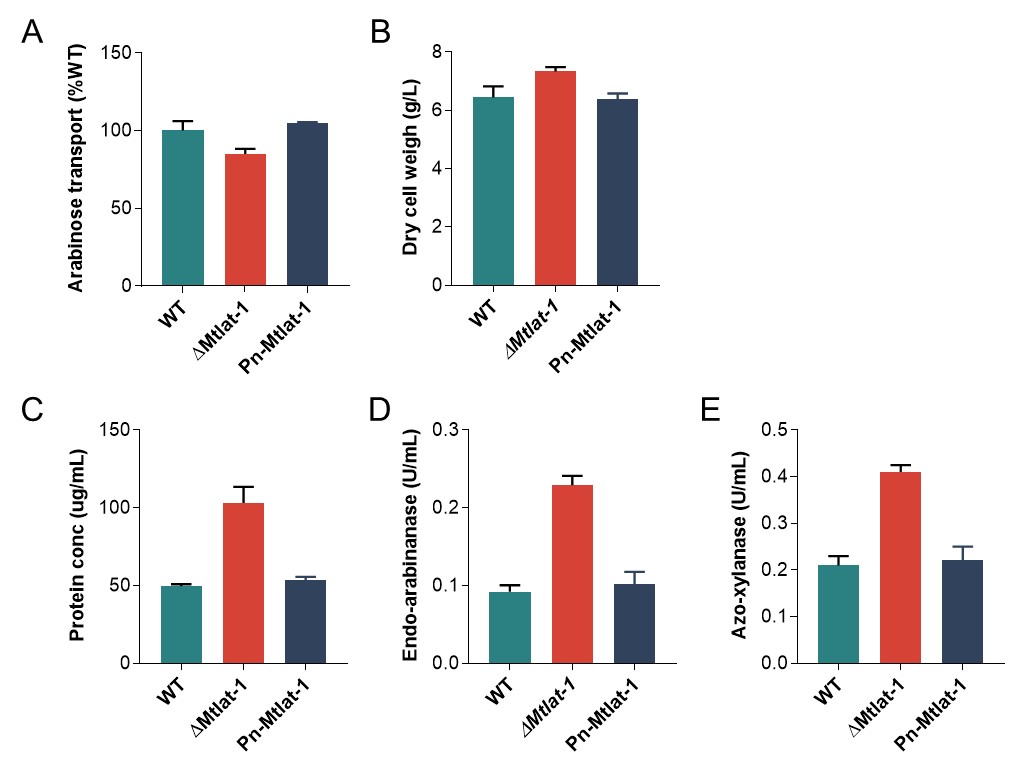
**

**Fig. S3** Growth phenotypes of the complementation strain of the Δ*Mtlat-1* mutant. (A) L-arabinose transport rates of mycelia from the complementation strain Pn-Mtlat-1. (B) Cell dry weight of the *M. thermophila* complementation strain after growth on 2% arabinan for 4 d. (C) Protein concentrations, (D) arabinanase activity, and (E) xylanase activity of the culture supernatants for *M. thermophila* grown in 2% arabinan medium. Error bars indicate the SD from at least three biological replicates.


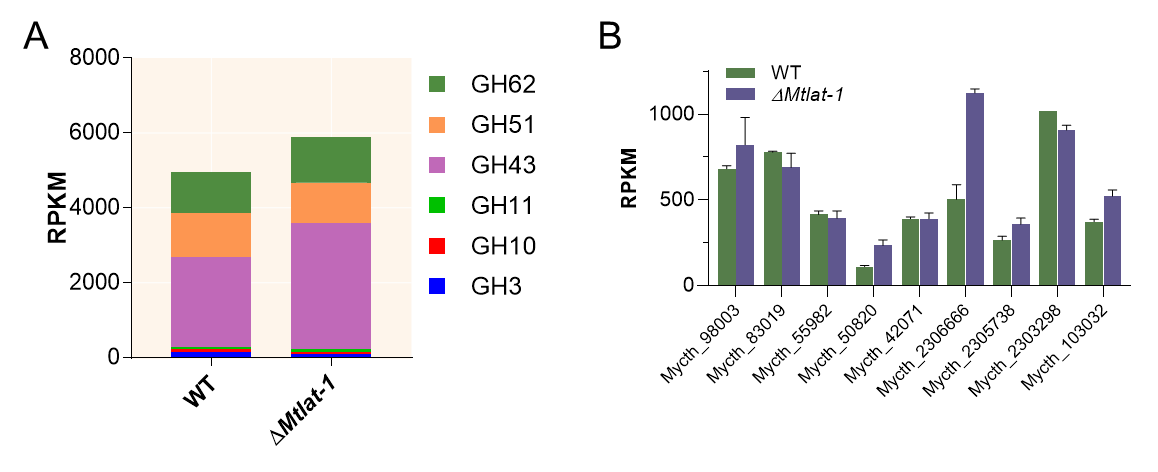


**Fig. S4** Comparative transcriptomic analysis of the WT and ΔMtlat-1 *M. thermophila* strains grown in arabinan medium for 2 d. (A) Total expression of genes encoding major hemicellulases from RNA-Seq data. (B) Transcriptional profiles of genes encoding arabinanolytic enzymes in the Δ*Mtlat-1* and WT strains when grown on 2% arabinan for 4 d.

**Fig. S5** Cell dry weight of *M. thermophila* strains after growth on 2% arabinan for 4 d.


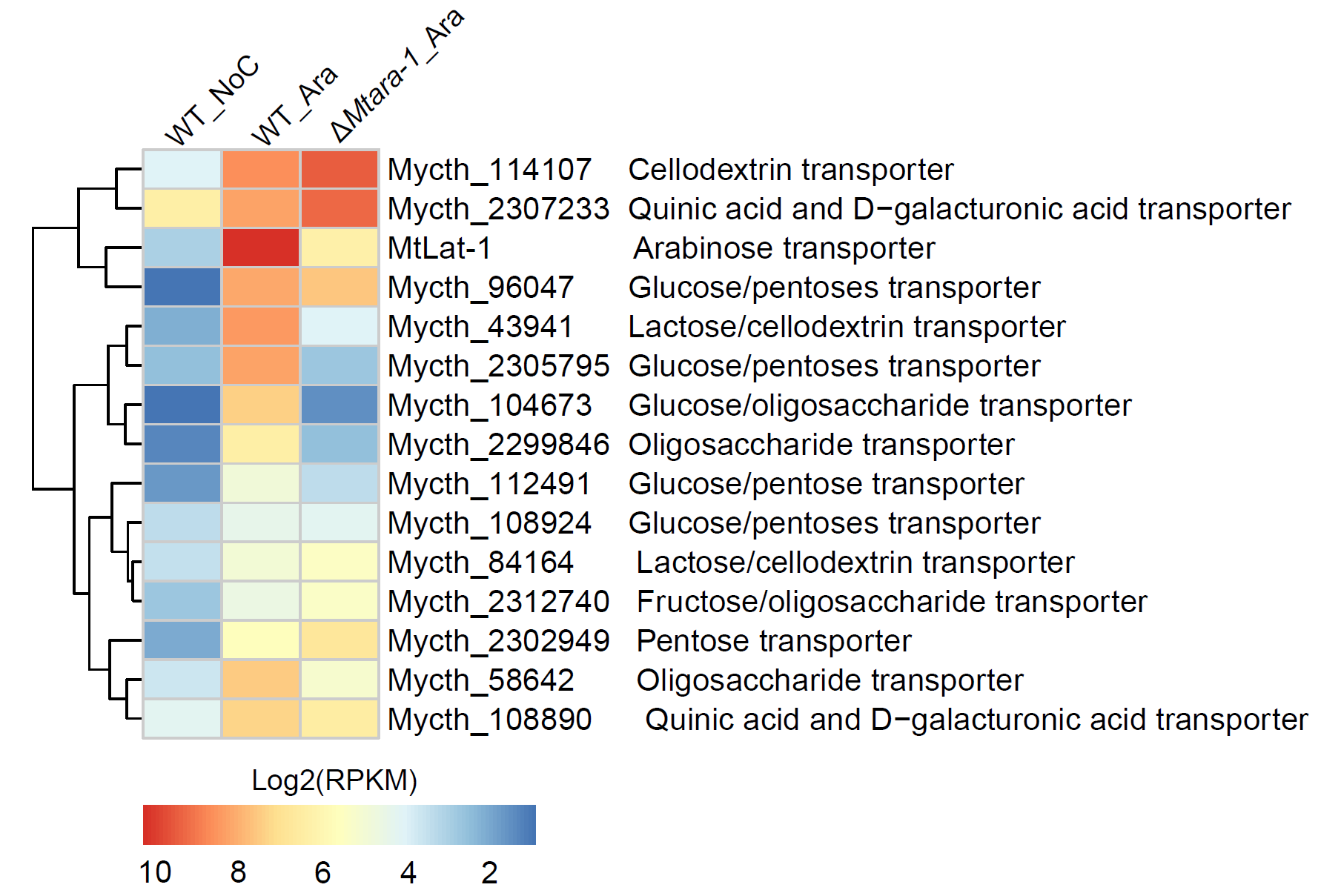


**Fig. S6** Heatmap analysis of expression profiles for putative sugar transporter genes with statistically significant differences in transcript levels between Δ*Mtara-1* and the WT under L-arabinose condition. Log-transformed expression values are color-coded.


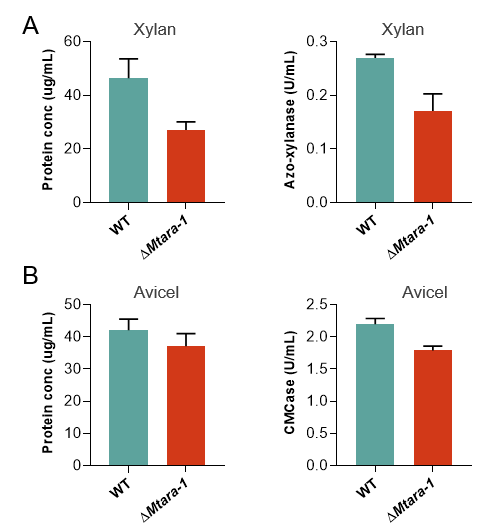


**Fig. S7** Protein concentrations and hemicellulase/cellulase activity of the culture supernatants for *M. thermophila* strain ΔMtara-1 grown in 1 × VMM with 2% xylan (A) or 2% Avicel (B) for 4 d.
